# Genetic profiling of *Mycobacterium bovis* strains from slaughtered cattle in Eritrea

**DOI:** 10.1371/journal.pntd.0006406

**Published:** 2018-04-17

**Authors:** Michael Kahsay Ghebremariam, Tiny Hlokwe, Victor P. M. G. Rutten, Alberto Allepuz, Simeon Cadmus, Adrian Muwonge, Suelee Robbe-Austerman, Anita L. Michel

**Affiliations:** 1 Department of Infectious Diseases and Immunology, Faculty of Veterinary Medicine, Utrecht University, Yalelaan 1, TD Utrecht, The Netherlands; 2 Department of Veterinary Sciences, Hamelmalo Agricultural College, Keren, Eritrea; 3 Agricultural Research Council-Onderstepoort Veterinary Research (ARC-OVR), Diagnostic Services Programme, Onderstepoort, South Africa; 4 Department of Veterinary Tropical Diseases, Bovine Tuberculosis and Brucellosis Research Programme, Faculty of Veterinary Science, University of Pretoria, Onderstepoort, South Africa; 5 UAB, Centre de Recerca en Sanitat Animal (CReSA, IRTA-UAB), Campus de la Universitat Autònoma de Barcelona, Bellaterra, Spain; 6 Departament de Sanitat i Anatomia Animals, Facultat de Veterinària, UAB, Bellaterra, Barcelona, Spain; 7 Department of Veterinary Public Health and Preventive Medicine, University of Ibadan, Nigeria; 8 Division of Genetics and Genomics, The Roslin Institute and the Royal (Dick) School of Veterinary Studies, University of Edinburgh, Easter Bush, Midlothian, United Kingdom; 9 National Veterinary Services Laboratories, United States Department of Agriculture, Ames, Iowa, United States of America; Hospital Infantil de Mexico Federico Gomez, UNITED STATES

## Abstract

*Mycobacterium bovis* (*M*.*bovis*) is the main causative agent for bovine tuberculosis (BTB) and can also be the cause of zoonotic tuberculosis in humans. In view of its zoonotic nature, slaughterhouse surveillance, potentially resulting in total or partial condemnation of the carcasses and organs, is conducted routinely. Spoligotyping, VNTR profiling, and whole genome sequencing (WGS) of *M*. *bovis* isolated from tissues with tuberculosis-like lesions collected from 14 cattle at Eritrea’s largest slaughterhouse in the capital Asmara, were conducted.The 14 *M*. *bovis* isolates were classified into three different spoligotype patterns (SB0120, SB0134 and SB0948) and six VNTR profiles. WGS results matched those of the conventional genotyping methods and further discriminated the six VNTR profiles into 14 strains. Furthermore, phylogenetic analysis of the *M*. *bovis* isolates suggests two independent introductions of BTB into Eritrea possibly evolving from a common ancestral strain in Europe.This molecular study revealed the most important strains of *M*. *bovis* in Eritrea and their (dis)similarities with the strains generally present in East Africa and Europe, as well as potential routes of introduction of *M*. *bovis*. Though the sample size is small, the current study provides important information as well as platform for future in-depth molecular studies on isolates from both the dairy and the traditional livestock sectors in Eritrea and the region. This study provides information onthe origin of some of the *M*. *bovis* strains in Eritrea, its genetic diversity, evolution and patterns of spread between dairy herds. Such information is essential in the development and implementation of future BTB control strategy for Eritrea.

## Author summary

The livestock sector plays a major role in poverty and hunger reduction in the vast majority of Africa, as a source of food, cash income, manure, draught power, transportation, savings, insurance and social status. However, for livestock to play this vital role, the impact of diseases of economic and zoonotic importance need to be reduced. Bovine tuberculosis, mainly caused by *Mycobacterium bovis*, is such an infectious disease. Slaughterhouse (gross pathology) surveillance, followed by bacterial culture and genotyping, are options to identify the disease-causing agents, their distribution, and enabling trace back of the sources of infections, in order to prevent their re-introduction and spread. Unfortunately, genotyping is by far not generally introduced in the continent. In the present study, tissues with tuberculosis-like lesions were collected from the Asmara municipal slaughterhouse, the largest slaughterhouse in Eritrea, and bacterial culture, classical *Mycobacterium tuberculosis* complex typing (Spoligotyping and VNTR profiling), as well as whole genome sequencing (WGS) were used to gain insight into the spatial and temporal distribution, genetic diversity and evolution of *M*. *bovis* strains circulating in Eritrean dairy cattle. The results revealed (dis)similarities of the Eritrean *M*. *bovis* strains with the strains generally present in Africa and Europe, potential routes of introduction to Eritrea and genetic diversity of the *M*. *bovis* strains. Future in-depth molecular studies including more samples from dairy cattle as well as cattle and goats from the traditional livestock sector are recommended.

## Introduction

*Mycobacterium bovis* (*M*. *bovis*) is the causative agent of bovine tuberculosis (BTB), achronic, infectious and contagious disease that also affects other domestic animals as well as humans [1,2]. Although BTB is prevalent in dairy cattle in Eritrea as shown by Omer et al. (2001) [3] and Ghebremariam et al. (2016) [4] by skin-test based survey, detection and isolation of the causative agent has never been done. Routine meat inspection at municipal slaughterhouses is performed for identifying tuberculosis-like lesions (TBL) that usually results in either total or partial condemnation of carcasses depending on the level of TBL dissemination, however, confirmatory testing or trace back epidemiological investigations are not conducted in Eritrea.Genotyping is a vital tool for trace back in epidemiological investigations, and according to Biek et al. (2012) [5] results from WGS alone can provide insight into TB epidemiology even in the absence of detailed contact data. Despite the usefulness of genotyping, it is rarely used in developing countries, i.e., in Africa, Asia, and South America [6–9]. The routine use of such tool in these countries could be instrumental in complementing BTB control strategies.

Spoligotyping and variable number of tandem repeat (VNTR) profiling have been used extensively in many countries to document the molecular epidemiology of *Mycobacterium tuberculosis* complex (MTBC)species [7, 10–14]. For this reason, the digital MTBC molecular genotypes are predominantly stored in these two forms globally[15–18].

The recent technological advancements in molecular genetics imply that we can now more than ever understand the molecular epidemiology of MTBC at amore granular level. In the last few years, whole genome sequencing (WGS) for typing of pathogens has been explored and yielded important additional information on strain diversity in comparison to the classical DNA typing methods. Analysis of data from WGS also allows detection of minute differences in genetic diversity and this has contributed retrospectively to outbreak investigations [19–23].Significantly, WGS allows for better genomic coverage withsingle nucleotide polymorphisms (SNP) profiling than the two classical typing methods [24,25]. WGS has also led to a significant growth in quantitative methodology that allows for a robust estimation of phylogenetic and temporal relationships between samples[26]. All these aspects are essential in enhancing our understanding of local and distant, recent and historical dynamics of BTB [5,24]. Although several reports predict that the use of WGS for genotyping will eclipse the classical MTBC typing tools [27], this will likely take longer to occurin Africa. It is therefore important to compare their utility in resource limited settings. Although such tools have never been used in Eritrea, their use would greatly enhance our understanding of: a) the genetic diversity of *M*. *bovis*, b) its evolution and c) the patterns of spread (spatial and temporal) between dairy herds, in the country and region. Such data (information) would be critical for safeguarding and further development of the dairy industry of Eritrea.In the present study,the classical MTBC typing tools (Spoligotyping and MIRU-VNTR) as well as WGS were used to gain insight into the spatial and temporal dynamics, genetic diversity and evolution of *M*.*bovis* strains circulating in Eritrean dairy cattle. Furthermore, to infer local and international historical phylogenetic relationships.

## Materials and methods

### Data and sample collection

Pooled tissue samples (lungs and pleura, mediastinal, bronchial, deep inguinal and lung lymph nodes), were collected from 15 animals that showed TBL in gross pathology, at the Asmara municipal slaughterhouse from March 2014 to May 2015. These 15 animals were all those with TBL during the study period. The animals were slaughtered for meat purpose and processed as part of the normal work of the abattoir. Approximately 5–10 grams of pooled tissues from each sampled animal were collected in sterile specimen containers, and immediately transported on icepacks to the National Animal and Plant Health Laboratory (NAPHL), Asmara, and stored at -20°C until processing for culture.

Data collected from individual animals(Table 1) included: source of the animal slaughtered,date of slaughter, species, breed, sex, age, pregnancy status (pregnant/non-pregnant), ante mortem clinical signs, post mortem lesions, and type of the tissue samples collected. In addition, retrospective meat inspection data for the period 2010 to 2015 were retrieved from the logbook of the slaughterhouse.

**Table 1:** Tissues with tuberculosis-like lesions (TBL) collected at the Asmara slaughterhouse, animals’ characteristics, ante mortem signs (AM signs), post mortem signs (PM signs), and origin of the slaughtered animals.

Date	TB number	Species	Breed	Sex	Age(yrs)	AM signs	PM lesions	Tissues collected	Origine of animals
28/03/14	TB8599	Bovine	HF*	F	5	Poor	TBL on lung and chest cavity	Lung and chest cavity tissues, bronchial and mediastinal. LN**	Asmara area
17/06/14	TB8600	Bovine	HF	F	7	Emaciated	TBL on chest cavity	Tissues with TBL from chest cavity	Asmara
9/04/14	TB8613	Bovine	HF	F	7	Normal	Traumatic pericarditis and TBL	Lung tissues and lung LN with TBL	Asmara area
10/11/14	TB8601	Bovine	local	M	7	Normal	TBL on peritoneum	Peritoneum and inguinal LN	Embaderho (Maekel)
19/11/14	TB8602	Bovine	HF	F	6	Emaciated	TBL on the chest	TBL from lung tissues and lung LN	Not available
24/11/14	TB8603	Bovine	HF	F	7	Emaciated	TBL on the chest& lung	Tissues of lung and sternum with TBL	Dekemhare (Debub)
29/12/14	TB8604	Bovine	HF	F	6	Normal	TBL on chest and lung	TBL from lung and chest	Not available
16/01/15	TB8605	Bovine	Cross	M	7	Normal	Abscess on chest and abdominal cavities	TBL from chest and abdominal cavity	Not available
2/10/15	TB8606	Bovine	HF	F	6	Normal	TBL in abdominal and chest cavity	Indguinal LN	Asmara
28/02/15	TB8607	Bovine	HF	F	4	Bloating	Lesions on abdominal and chest cavity	TBL from chest and abdominal cavity	Asmara area
20/04/15	TB8608	Bovine	HF	F	5	Normal	TBL in body cavity	Inguinal and sternal LN	Asmara
18/05/15	TB8609	Bovine	HF	F	4	Normal	Miliary TBL	Pleural and deep inguinal LN	Asmara
19/05/15	TB8910	Bovine	HF	F	8	Emaciated	Few TBL on the chest	Lung and bronchial LN	Asmara Unaminassie
23/05/15	TB8611	Bovine	HF	F	7	Normal	TBL on chest cavity	TBL from pleura and mediastinal LN	Not available
30/05/15	TB8612	Bovine	Cross	F	6	Normal	Hyperemic lesions on pleura and its cavity	TBL from chest, inguinal and bronchial LN	Not available

*HF = Holstein-Friesian

**LN = lymph nodes

### Isolation and identification of *Mycobacterium bovis*

Samples were processed for *M*. *bovis* culture as follows: approximately 5 g of each pooled tissue sample with TBL per animal was cut into small pieces and covered with 100 ml of sterile distilled water in a biohazard cabinet (Esco Class II BSC; Labotec, SA). The samples were homogenized using an Ultra-Turrax homogenizer at 17500 rpm (Separation Scientific, SA). Seven millilitres of the homogenate was poured into each of two separate 15 ml falcon tubes, and the remaining homogenate was poured into individual 50 ml centrifuge tubes and stored at -20°C as reference samples. The samples were decontaminated with 7 ml of 2% HCL (final concentration of 1%) and 7 ml of 4% NaOH (final concentration of 2%), respectively, and incubated at room temperature (18–25°C) for 10 min. After subsequent centrifugation (HeraeusLabofuge 400) of the samples at 3500 rpm for 10 min., supernatants were poured off and 7 ml of sterile distilled water was added. After washing, the centrifugation step was repeated and most of the supernatant was poured off. The pellets were re-suspended in a volume of approximately 1ml using a sterile inoculation loop. Two loops of each of the pellets were spread evenly onto two Löwenstein-Jensen (L-J) media slants supplemented with pyruvate (National Health Laboratory Service, SA) and onto one L-J medium slant supplemented with glycerol (BD Diagnostics), and incubated at 37°C for up to ten weeks. The slants were monitored weekly for mycobacterial growth.

Ziehl-Neelsen staining was conducted andlysate (DNA) of acid fast bacteria was subjected to polymerase chain reaction (PCR) testing to identify bacteria as MTBCas previously described [28,29]. Subsequently, deletion analysis was performed on the isolates using PCR primers targeting the RD4 (region of difference-4) as previously described for *M*. *bovis* identification [30].

#### Genotyping

Genotyping was conducted first using the standard, spoligotyping and VNTR profiling methods, followed by bioinformatics tools as described below to analyse the WGS data.

#### Spoligotyping

Spoligotyping was conducted according to previously used standard methodology [14] using a commercial kit (SPOLIGO TB, Mapmygenome, India),*M*. *bovis* BCG and distilled sterile water were used as positive and negative controls, respectively. Briefly, DNA samples from fresh isolates of the identified MTBC, confirmed through deletion typing, were used. The direct-repeat (DR) region was amplified with primers DRa (biotinylated) and DRb, and the amplified DNA was hybridized to inter-DR spacer oligonucleotides covalently bound to a membrane.

#### Variable Number of Tandem Repeat (VNTR) typing

PCR amplification of DNA for VNTR typing was performed using a set of 13tandem repeat loci recently identified as stable and polymorphic for South African *M*. *bovis* isolates [11]. These included the (4) ETRs loci, (4) QUB loci, (3) MIRU and (2) Mtub loci (i.e. ETR-A, -B, -C, and -E; Qub-11a, -11b, -18 and -26, MIRU 16, 23 and 26, as well as Mtub 12 and 21). The loci were amplified individually as previously described [31]. The band sizes were converted into number of tandem repeats at each locus based on the allele naming table provided [31].

### Identification of *M*. *bovis* clonal complexes

The three features used to distinguish *M*. *bovis* clonal complexes were: a) they are a derivative of most recent clonal ancestors (MRCA) spoligotype b) region of difference deletion and c) geographic restriction (Example: African 1 is localized in West Africa)

#### a) Clonal complexes African 1 and 2

The status (presence or absence) of the regions of difference for African 1 and 2 (RDAF1& RDAF2)in the isolates was assessed by multiplex PCR following procedures described earlier by Müller et al. [17], and Berg et al. [16]with minor modifications (2 μl of DNA template was used to make a final reaction volume of 21 μl each), respectively.

#### b) Clonal complexes European 1 and 2

The status of the European 1 region of difference (RDEu1) was determined by PCR using two primers targeting the flanking regions of the Eu1 deletion boundary as previously described by Smith and co-workers [18].Whereas, the status of the European 2 region of difference (RDEu2) was determined by performing a PCR restriction endonuclease analysis to determine the presence of the SNP in *gua*A gene[15].

### Whole genome sequencing (WGS)

To obtain the whole genome sequences, DNA of the 14 Eritrea *M*. *bovis* isolates was extracted (dx.doi.org/10.17504/protocols.io.nsgdebw) and sequenced on a MiSeq instrument (Illumina, San Diego, CA) using 2x250 paired-end chemistry and the Nextera XT library preparation kit (Illumina, San Diego, CA). FASTQ files from the instrument were put through the National Veterinary Services Laboratories (NVSL)in-house pipeline (see https://github.com/USDA-VS). Briefly, reads were aligned to the reference genome AF2122/97, NCBI accession number NC_0002945, using BWA and Samtools[32,33]. A depth of coverage of 80X was targeted. BAM files were processed using Genome Analysis Toolkit (GATK)’s best practice workflow. SNPs were called using GATK’s HaplotypeCaller outputting them to variant call files (VCF)[34–36]. Results were filtered using a minimum QUAL score of 150 and AC = 2. From the VCFs, SNPs gathered were outputted to three formats: an aligned FASTA file; tab-delimited files sorted by position location and by SNP groups; and a maximum likelihood phylogenetic tree created with RAxML[37]. The tree was built using a GTR-CAT model with input taken as an alignment file containing only informative and validated SNPs. SNPs were visually validated using Integrative Genomics Viewer (IGV) [38]. Because WGS isolates from this region of the globe are not readily available, databases from three laboratories (United States Department of Agriculture, Centre de Recercaen Sanitat Animal (CReSA)—Institute de Recerca i Technologia Agroalimentáries (IRTA), Spain, and Tuberculosis Research Laboratory, Department of Veterinary Public Health and Preventive Medicine, University of Ibadan, Nigeria) that are actively sequencing *M*. *bovis* isolates were queried and field isolates that were within 150 SNPs of the Eritrea isolates were included in our analysis. Also included for perspective were widely available reference strains, AN5, Ravenel, 95–1315, AF2122/97, BCG, and BZ-31150. “FASTQ files from the isolates sequenced were uploaded into NCBI short read archive. Accession numbers Bioproject and sample numbers are listed in supplemental S1 Table.

## Results

### TBL detection at meat inspections

During the period 2010 to 2015, 78,820 cattle were slaughtered and 38 carcasses, originating from Maekel and Debub regions, were totally condemned due to generalized TBLs showing caseous necrosis identified in gross pathology in the lungs, livers, pleura (chest), peritoneum, and lymph nodes. Besides, fore quarters of three animals, plucks, shoulders, chests and heads of six cattle were partially condemned due to the presence of TBL (Table 2). All, except one (local breed), of the condemned carcasses were of the exotic HF breed or their crosses.

**Table 2:** Total number of cattle slaughtered, and number of totally and partially condemned carcasses and organs due to the presence of tuberculosis-like lesions (TBL) from 2010 to 2015 (inclusive) at Asmara municipal slaughterhouse in Eritrea.

Year	Number of cattle slaughtered	Number of carcasses totally condemned	Body parts and organs partially condemned	Number of animals	Cattle breeds and sex
2010	14,919	6	Fore quarters, pluck (thoracic viscera and liver) and chest	1	Exotic (HF*), male
2011	11,976	8	Fore quarters, plucks, heads and shoulders	2	Exotic (HF), females
2012	12,307	5	Head, plucks and shoulders	3	Exotic(HF), 1 male and 2 females
2013	13,018	10	Heads, Plucks and shoulders	3	Exotic (HF), females
2014	13,359	1	Plucks and shoulders	1	Local, male
2015	13,244	8	Plucks and shoulders	2	Exotic (HF), females

*HF = Holstein-Friesian

Out of the 15 animals sampled from March 2014 to May 2015, nine originated from Maekel and one from Debub, while the origin of the other five slaughtered animals was unknown due to lack of records. Detailed gross pathology information on the tissues collected is presented in Table 1. During this period 26,603 cattle were slaughtered and nine out of the 15 carcasses sampled, were totally condemned due to generalized TBL. In addition, the entire plucks and shoulders of three animals were partially condemned (Table 2), and from three other animals, tissues with TBL were collected and the carcasses passed for consumption.

### *Mycobacterium bovis* culture and identification

Out of the 15 pooled tissue samples cultured on L-J media slants supplemented with pyruvate, 14 yielded smooth dysgonic growth, suggestive of *M*. *bovis* presence. All the 14 isolates were identified as MTBC. Subsequent examination by *M*.*bovis* specific PCR targeting the RD4, yielded banding patterns typical of *M*. *bovis* with a 268 bp product indicating RD4 deletion.

### *Mycobacterium bovis* PCR based genotyping

#### Spoligotyping

**Fig 1:**
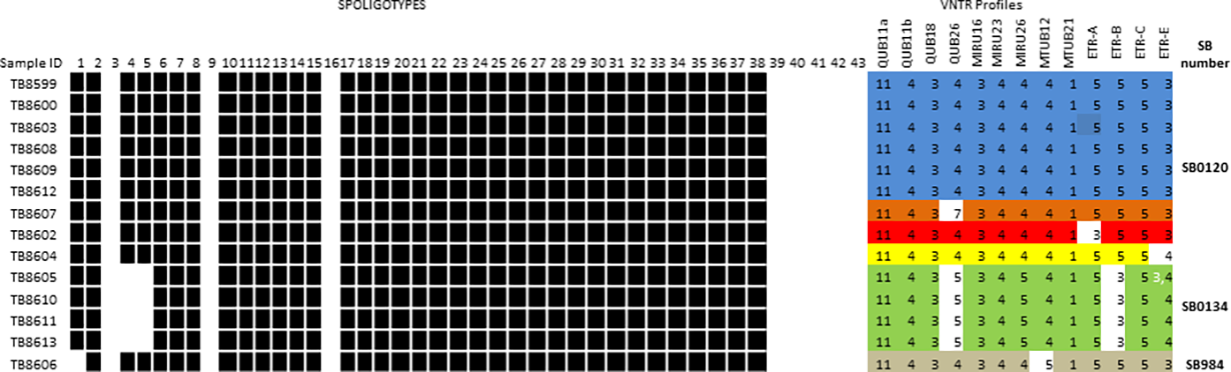
Spoligotype patterns with their SB-numbers retrieved from www.*Mbovis.org* of 14 *M. bovis* isolates (TB numbers) and VNTR profiles with their designations (ER-1 -ER-6) from tissues with TBLs collected at the Asmara municipal slaughterhouse.

The spoligotyping resulted in 3 distinct spoligotype profiles (Fig 1). The predominant spoligotype was SB0120 (9/14; 64%), characterized by the absence of spacers 3, 9, 16, and 39–43; followed by SB0134 (4/14; 29%), that showed absence of spacers 4 and 5 in addition, and lastly SB0948 (1/14; 7%); with the absence of spacers 1, 3, 9, 16, and 39–43. Designations for the spoligotypes corresponding to the spoligotype profiles in our isolates were obtained from http://www.*M.bovis*.org database.

#### VNTR typing

From the 14 *M*. *bovis* isolates, VNTR typing using a 13-loci VNTR panel revealed six VNTR profiles. Within the strains analyzed, only VNTR loci QUB26, ETR E, B, and MIRU 26 showed variations amongst the isolates, whereas the remaining loci (ETR A, C, Qub11a, 11b, 18, MIRU 16, 23, Mtub16 and 21) were monomorphic (Fig 1). One isolate exhibited two different VNTR alleles (3 and 4 tandem repeats) for locus ETR-E. For convenience reasons, the six VNTR profiles found were designated: VNTR profiles-ER-1 to -ER-6.

VNTR profile-ER-1 shared by six isolates (TB8599, TB8600, TB8603, TB8608, TB8609, and TB8612) was the most common one;VNTR profile-ER-5 was shared by 4 isolates (TB8605, TB8610, TB8611, and TB8613); VNTR profile-ER-2, -3–4 and -6, were represented by one isolate each (TB8607, TB8602, TB8604, and TB8606, respectively). Four VNTR profiles (ER-1 to ER-4) corresponded to the SB0120 spoligotype, and two VNTR profiles, i.e., ER-5 and ER-6 corresponded to the SB0134 (TB8605,TB8610,TB8611,TB8613), and SB0948 (TB8606), respectively (Fig 1). The spoligotype SB0948 was clustered within the SB0120 group in its VNTR profile, with only one locus (Mtub21) difference from the rest of the group (Fig 1).

#### Clonal complex characterization

None of the 14 *M*. *bovis* isolates belonged to the RDAf1 (PCR product size of 350bp); RDAf 2 (PCR product size of 458bp), RDEu 1 (PCR product size of 1206bp), and RDEu 2. The spacers known to be deleted in the respective clonal complexes (i.e. spacer 30 in Af1, spacer 3 to 7 in Af2, spacer 11 in Eu1, and spacer 21 in Eu2) are intact in the Eritrean strains. The *M*. *bovis* positive control used (South African isolate; TB8569) was Eu1 clonal complex that demonstrated intact RDAf1 and 2, spacer 21, and the absence of spacer 11. Of the 14 *M*. *bovis* isolates, two isolates (TB 8603 and 8613) were found to have the guaA mutated as indicated by the presence of the SNP leading to a single band of 179 bp following a PCR-restriction endonuclease analysis conducted to determine the presence of the SNP in guaA. The absence of a SNP in the guaA gene was demonstrated by two bands of 145 and 34 bp.

#### *Mycobacterium bovis* SNP based genotyping and phylogenetic relationships

**Fig 2:**
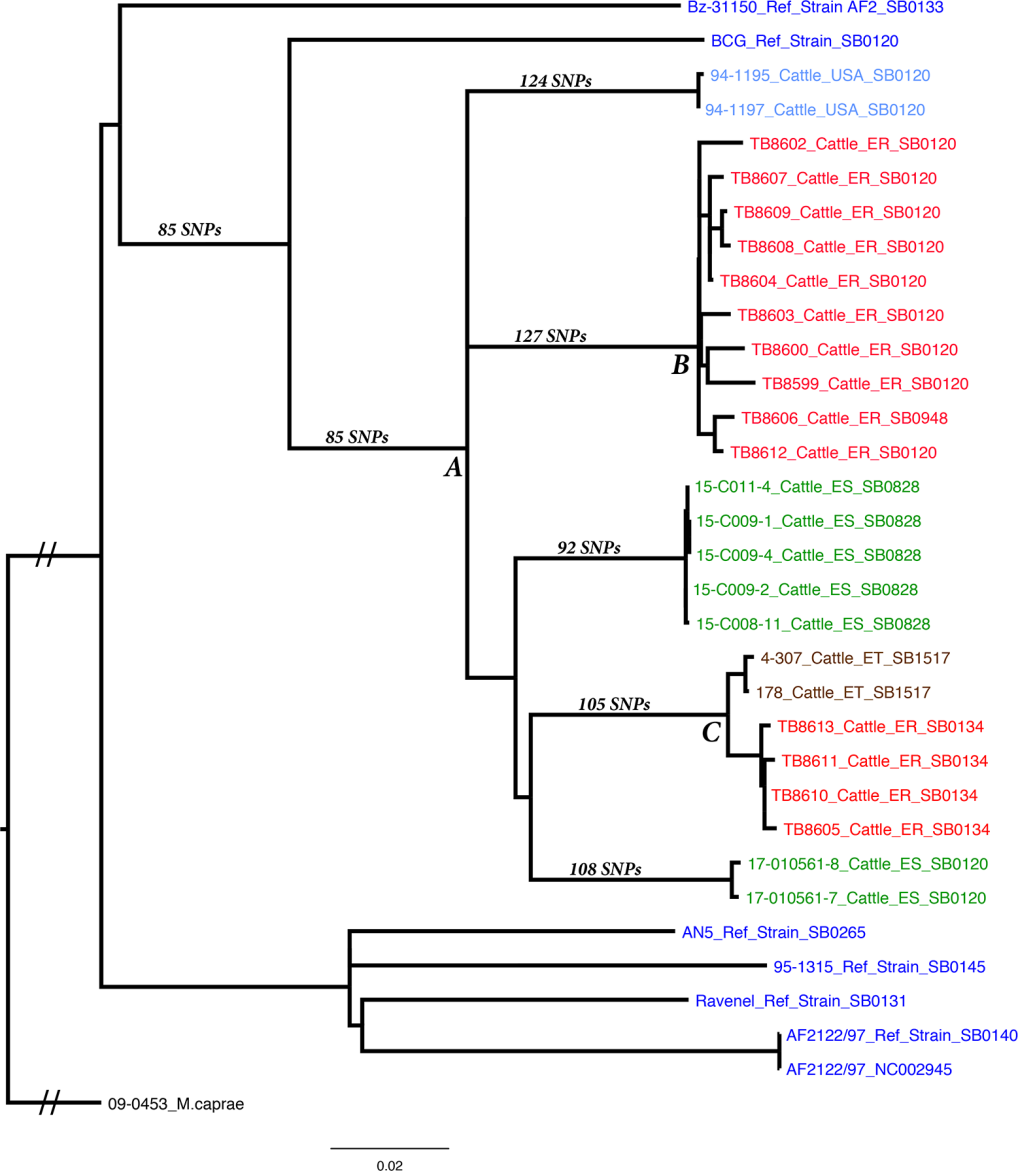
Whole genome sequence phylogenetic tree created using RAxML of 14 Eritrea dairy cattle abattoir samples and other field isolates that happen to share the same common ancestor with Eritrean isolates in the National Veterinary Services Laboratories’ database. These include cattle isolates from Ethiopia, United States and Spain. Also included in the tree are well known type strains, including BCG, Bz-31150 –a recently sequenced AF2 strain, AN5 –used in PPD production, 95–1315 –Michigan deer strain, Ravenel and AF2122/97.

WGS and SNP analysis (Fig 2), shows that the Eritrean isolates clustered into two distantly related groups, containing an additional 135–159 SNPs since sharing a common ancestor (Labeled A in Fig 2) along with isolates from Spain and the USA, respectively. The Eritrean cluster consisting mostly of SB0120 isolates which were more diverse than those in the SB0134 cluster i.e. a SNP difference ranging from 8–30 from a common ancestor(Labeled B in Fig 2). There were also sub-clusters within this group; 5 isolates were only 5–6 SNPs from a common ancestor.Eritrean samples with the SB0134 spoligotype were only 10 SNPs from a shared common ancestor (Labeled C in Fig 2) with two isolates from Ethiopia. Interestingly the four Eritrean isolates were within a distance of 5–6 SNP from each other. Supplemental S2 Table contains the location and annotation of each SNP identified in the sequences from Fig 2. The overall phylogenetic structure of *M*. *bovis* isolates in the NVSL database are shown in Supplemental S2 Fig.

## Discussion

Livestock production in general (‘intensive dairy’ and extensive traditional livestock rearing) is the main stay of the vast majority of people in Africa. In the Horn of Africa, where Eritrea is located, about 41 million people keep some livestock as a source of food, cash income, manure, draught power, transportation, savings, insurance and social status [39]. Hence, livestock plays a major role in poverty and hunger reduction. For livestock to play its crucial role in achieving food security and become economically viable in Eritrea, the impact of major transboundary diseases needs to be reduced. One of these diseases is BTB. Genotyping plays vital role in highlight of transmission networksof pathogens and enables trace back sources of infections, in order to prevent their re-introduction and spread. For this purpose, classical and state-of -the-art genotyping tools were used in the present study of *M*. *Bovis* isolates circulating in dairy cattle in Eritrea.

### *Mycobacterium bovis* PCR based genotyping

The dominant spoligotype identified in our study was SB0120, named BCG-like by Haddad et al. [13] and considered as parental strain for the *M*. *bovis* vaccine strain. It accounted for 64% of the isolates, whereas the other two spoligotypes SB0134 and SB0948 did so for 29% and 7%, respectively. The first two strains are widely distributed in a number of African countries, namely; Ethiopia, Algeria, Zambia, South Africa [6,10,16,40–43] as well as in Italy, Spain, other European countries and Mexico [13,44–49]. In addition to cattle, SB0120 affects wildlife and humans in Africa and Europe [50–53]. The third spoligotype(SB0948) has been reported in France, Italy, and in Zambia [13,41,44].

The relatively high frequency of the spoligotype SB0120 found in the present study may indicate its predominance in Eritrean dairy cattle, though difficult to conclude with such small sample size. The second predominant spoligotype (SB0134) appears to have evolved from spoligotype SB0120 by the loss of spacers 4 and 5 in addition to spacers 3, 9, 16, and 39–43 that classify spoligotype SB0120. This finding might not be surprising, in view of the past trade relations between Eritrea and Ethiopia, as both SB0120 and SB0134 spoligotypes are also present in Ethiopia. Besides, these two countries share open borders that consequently allowed the uncontrolled movement of animals as obtained in most African countries. Therefore, it might be plausible to speculate that these strains of *M*. *bovis* are shared between Eritrea and Ethiopia. On the other hand, it might also be plausible to suggest Italy as a possible source of these strains, on the following grounds: a) long historical ties (1900 to 1970s) between Eritrea and Italy existed, b) Italian settlers initiated the establishment of dairying in Eritrea in the 19^th^ century by importing exotic breeds(Holstein–Friesian), c) the *M*. *bovis* spoligotypes detected in our study are also wide spread in Italy. Although the reason for the apparent predominance of the two spoligotypes (SB0120 and SB0134) needs further study, as this may indicate an epidemiological link between different dairy farms/regions in Eritrea, as buying and selling of cows between dairy farms is common in the country[54] without following strict sanitary rules.Since not all the slaughtered cattle with TBL had records of their farm of origin, it may also be possible to suspect that some of the slaughteredanimals might have originated from the same farm. It is noteworthy, however, that based on the WGS data there appears to be at least two introductions of *M*. *bovis* into Eritrean dairy cattle, an SB0120 strain and SB0134 strain. The SB1517 (Ethiopian strain; Fig 2) is an offspring of SB0134 suggesting that the common ancestor of the cluster was SB0134.

**Fig 3:**
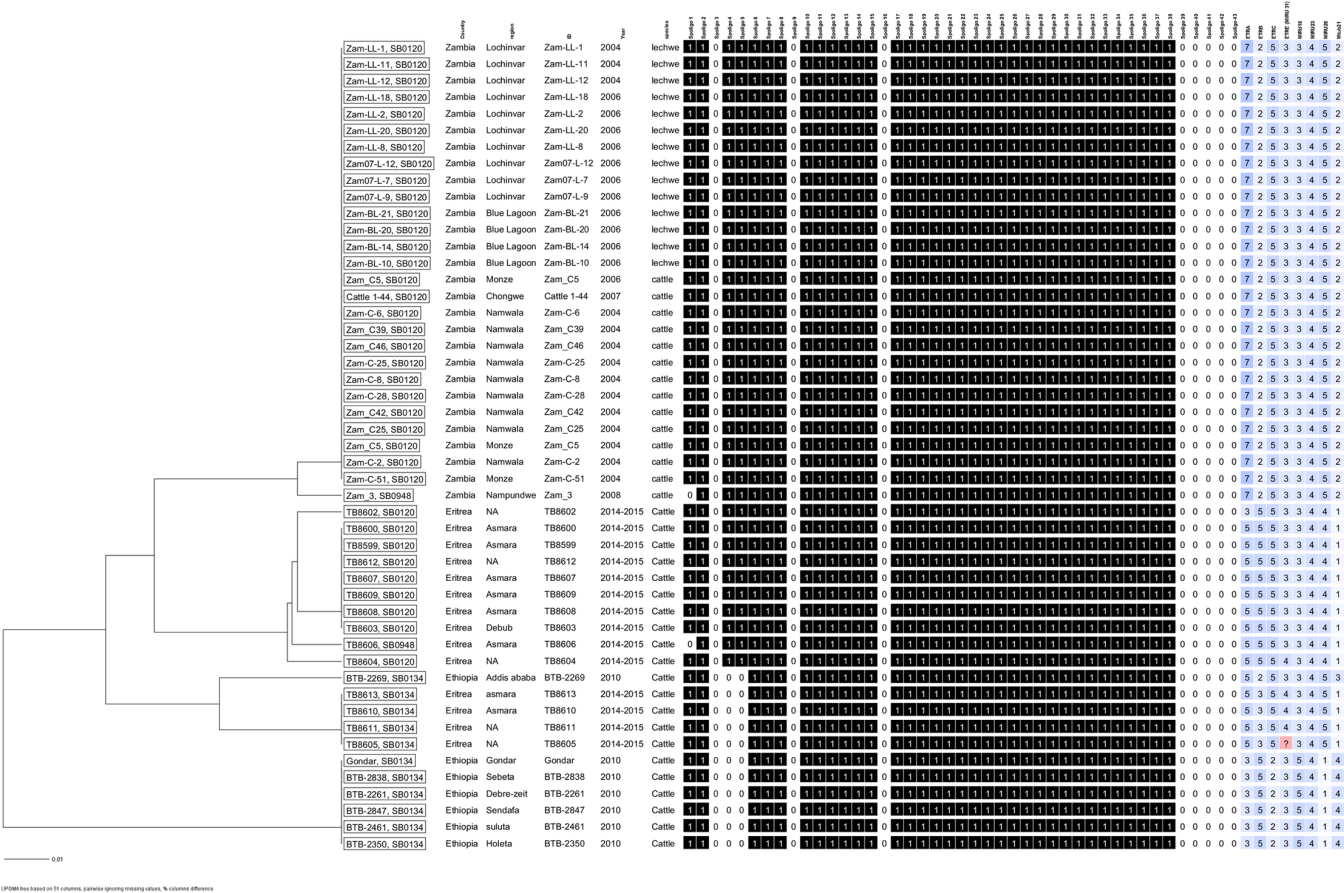
African comparison of spoligo patterns and VNTR profiles showing common traits with the Eritrean samples.

Spoligotype SB0948 was found in only one animal. It is a descendant of spoligotype SB0120 as it differs by the absence of spacer 1 only, and deviates only by the Mtub21 locus in its VNTR profile from the other members of the SB0120 group (Fig 1). Further, the WGS data confirmed that SB0948 is a recent descendant of a sub-cluster of SB0120 isolates. Though unclear what its relevance is in neighboring Ethiopia, this spoligotype was reported in several countries in Africa and Europe [13,41,44,48,55].The African and global comparisons of spoligotype profiles (Fig 3and S1 Fig)demonstrated the regional and global distribution of the spoligotype and VNTR profiles and their similarities with the Eritrean ones. These similarities could be attributed to the following two plausible reasons: a)inter-regional and global livestock trade, b) colonial livestock and livestock product trade within their then colonies and outside.

Variable number tandem repeat (VNTR) profiles are considered appropriate to complement spoligotyping due to their ability to discriminate between *M*. *bovis* strains as defined by spoligotyping[15,55,56].The three spoligotypes were clustered into six VNTR profiles (Fig 1). The diversity seen in the VNTR profiles may suggest that *M*. *bovis* has been circulating in the dairy herds of the country for quite a long time with only minor mutations as the BCG-like spoligotype (SB0120) is the predominant one. Four of the VNTR profiles (ER-2, -3, -4 and 6) may have derived from the predominant VNTR profile ER-1, that corresponds to spoligotype SB0120. According to Smith et al.[49], strains bearing the same spoligotype pattern are assumed to be a set of individuals derived relatively recently by clonal replication from a single ancestral cell. On the basis of the VNTR profile, both strains, SB0948 and SB0134, are clustered within the SB0120 group with a loss of only one locus (Mtub12) in the former and two loci (ETR-B and ETR-E), in the latter strain, respectively. One of the VNTR profiles within the SB0134 strain exhibited two different VNTR alleles (3 and 4 tandem repeats) for locus ETR-E (Fig 1), suggesting either a mixed infection with two distinct strains or a microevolution in this strain. The VNTR profiles found in our study showed clonal variants differing at their loci as compared to what was reported in other parts of Africa (i.e., Zambia) (Fig 1 & Fig 3), though they were all *M*.*bovis* strains belonging to SB0120 spoligotype. This clonal difference (Fig 1 & Fig 3) seen in our study may have been attributed to the absence of active livestock (dairy cattle) trade between Eritrea and other parts of Africa (Zambia) or due to the different geographical locations and livestock management systems between the countries, that might have dictated the microevolutions (mutations) differently. The possible reason for having the same spoligotype (SB0120) in Eritrea and other African countries (S1 Fig), might be that the source of the cattle for Eritrea and the other countries was Europe, as Europe is the source for the high yielding dairy cows, like the Holstein Friesians, that are imported by most African countries with the aim of improving milk production in their countries in order to realize food security.

### Clonal complex characterization

The investigation of the 14 *M*. *bovis* isolates for clonal complex differentiation revealed that they belonged to none of the complexes identified so far i.e., African 1(RDAf1), African 2 (RDAf2), European 1 (RDEu1) and European 2 (RDEu2)[15–18]. The absence of members that belong to clonal complex African 1& 2 in our samples could suggest limited introduction of such strains from the neighboring Eastern and Western African livestock movement routes. It is noteworthy, that these two strains (SB0120 and SB0134) are present in Ethiopia [16], although most of the other strains in this country belong to Af2. In the current study, little strain diversity is recorded (Fig 1)as compared to studies conducted in other countries with similar agricultural setting like Eritrea[6,42].

### *Mycobacterium bovis* SNP base genotyping and phylogenetic relationships

The WGS results matched the conventional laboratory methods with better resolution. These data support two separate introductions of *M*. *bovis* into Eritrea, with subsequent localized spread. The common ancestor of these two groups is shared widely with isolates in the USA and Spain, with greater diversity found in Spain suggesting an introduction from Europe.

The presence of a common ancestor in these distantly located countries may be due to the international livestock trade between these countries, geographical proximity and similar livestock production systems. Example: the origin of the high yielding dairy breed (Holstein Friesian) is Europe. As indicated in the spoligotyping section above, the spoligotype SB0120, predominant in our study, is also ubiquitous in Europe, especially in France[13], Italy [44], Portugal[45], and Spain[48], most likely as a result of geographical closeness and trade relations between these countries. Therefore, our finding may not be a surprise, given the historical establishment of ‘intensive’ dairy farming by the Italian settlers in Eritrea through the importation of high yielding dairy breeds (Holstein Friesians) to meet the high demand for milk and dairy products. The fact that the Eritrean strains are between (close to) Spain samples (Fig 2)may suggest two introductions or may be just one introduction; i.e., from Europe (Italy). Since we do not have information that shows historical, political or trade ties between Spain and Eritrea, we can speculate that either the strains are circulating in Italy and Spain. Or that, the Italian settlers during the establishment of dairy farms may have imported the cattle from Spain or other European countries where the same strains of *M*. *bovis* might have been circulating. A classical analogy for this speculation may be rinderpest that was brought to Sub-Saharan Africa by Italian forces in 1889, with infected cattle they had imported from India, Aden, South Russia to feed their army that had then occupied Massawa (Eritrea) [57]. However, although phylogenetic comparison with Italian *M*. *bovis* isolates could not be done in our study, we cannot refute the possibility that these strains originate from Italy or via the above indicated routes from other countries.

The second probable route of introduction for one of the groups of the Eritrean strains, but not for the other, may be Ethiopia considering the long and close historical relationship and uncontrolled livestock movement between these two countries. The Ethiopian and Eritrean samples have accumulated 8–16 additional SNPs since sharing a common ancestor suggesting a recent common source and regional spread. But the four Eritrean samples (strains) are within 5–6 SNPs from sharing a common ancestor suggesting these isolates have established and spread within Eritrea, though it might be premature to reach into conclusion with such small sample size. Eritrea, on the other hand, might have introduced this strain to Ethiopia.This is plausible because both intensive dairy farming, established 100 years ago in Eritrea and the first report of BTB (Pirani, 1929), cited by Omer et.al. [3],occurred earlier than in Ethiopia where ‘intensive’ dairy farming started in the 1950s (1947) by importing Friesians and Brown Swiss [58]. This was followed by the detection of acid fast bacilli in a cow’s milk, in one study, and detection of what was called ‘*Mycobacteria tuberculosis* bovine type’ seemingly, *M*.*bovis* from 18 cattle, in another study, in Eritrea, by Sfroza in 1944[3].

The samples collected in this study are not considered representative of all strains possibly circulating in Eritrea. However, Asmara slaughterhouse, as the country’s biggest facility mostly slaughters exotic cattle breeds from various regions in Eritrea in which previously a high BTB prevalence was reported [4]. Therefore, the panel of samples still provides a valuable insight in the genetic strain composition from mostly dairy producing regions in Eritrea and a valuable basis for future investigations.

The current study characterized strains of *M*. *bovis* in Eritrea and revealed their (dis)similarities with the strains generally present in Africa and Europe, as well as potential routes of introduction of *M*. *bovis*. Though the sample size is small, our study provides important information as well as availability of technology for future in-depth molecular studies including more samples from dairy cattle as well as cattle and goats from the traditional livestock sector.This study provides information on the origin of the *M*. *bovis* strain in Eritrea, its genetic diversity, evolution and patterns of spread (spatial and temporal) between dairy herds. The information obtained will be instrumental in making informed decisions in future BTB control strategy for Eritrea.

### Limitations

Our study has some limitations. The samples were collected from one slaughterhouse and were few due to the absence of tissues with TBL during the study period. The low prevalence of BTB in the traditional livestock raising system [59] where majority of slaughtered animals come from, has limited the possibility of detecting more *M*. *bovis* strains from different geographical regions of Eritrea.

### Conclusion and recommendations

Genetic profiling of *M*. *bovis* strains is a highly useful approach which can aid in the study and control of the temporal and geographical disease spread in the country and the African continent where BTB is largely uncontrolled.We recommend future studies in Eritrea to include genetic profiling of Italian isolates so as to support or negate our hypothesis with certainty than just live with speculation that the origin of the Eritrean *M*. *bovis* strains was Italy.

In future studies in Eritrea, inclusion of more regional slaughterhouses including animal traceability will enable us gain greater insight into the epidemiology of BTB in the country which will allow the *M*. *bovis* genotype to be linked to the population from which it was obtained.

We also recommend that simultaneous detection and strain differentiation of *M*. *bovis* isolates should become a reality in the routine of human tuberculosis reference laboratories, as well as in the routine meat inspection at municipal slaughterhouses. Therefore, using the One Health paradigm (i.e. interdependence between the medical and veterinary fields), greater integration between agriculture and health sectors could be an important strategy to control *M*. *bovis* in several places in the world where the agent is disseminated between animals and humans.

## Supporting information

S1 Table Accession numbers, Bioproject and sample numbers in “FASTQ” files from the isolates sequenced and uploaded into NCBI short read archive. The Eritrean *M*. *bovis* isolates are compared with the widely available *M*. *bovis* reference strains, the field strains from the collections of United States Department of Agriculture, Centre de Recercaen Sanitat Animal (CReSA)—Institute de Recerca i Technologia Agroalimentáries (IRTA), Spain, and University of Ibadan, Nigeria, that shared the same common ancestor.
(XLSX)

S2 Table SNP table comparing the Eritrean strains with Ethiopian, Spanish and American (USA) strains with reference to Bz-31150_Ref_Strain AF2_SB0133 and BCG_Ref_Strain_SB0120. (XLSX)

S1 Fig Global comparison of spoligotypes in relation to the spoligotypes of the Eritrean *M*. *bovis* isolates. (TIF)

S2 Fig The overall phylogenetic structure of *M*. *bovis* isolates in the NVSL database with the Eritrean and related strains. (TIF)
