# Supplementary figures and images for: Genetic profiling of Mycobacterium bovis strains from slaughtered cattle in Eritrea
Source: PLoS Negl Trop Dis. 2018 Apr 17;12(4):e0006406. doi: 10.1371/journal.pntd.0006406 (PMC5922621; doi:10.1371/journal.pntd.0006406)

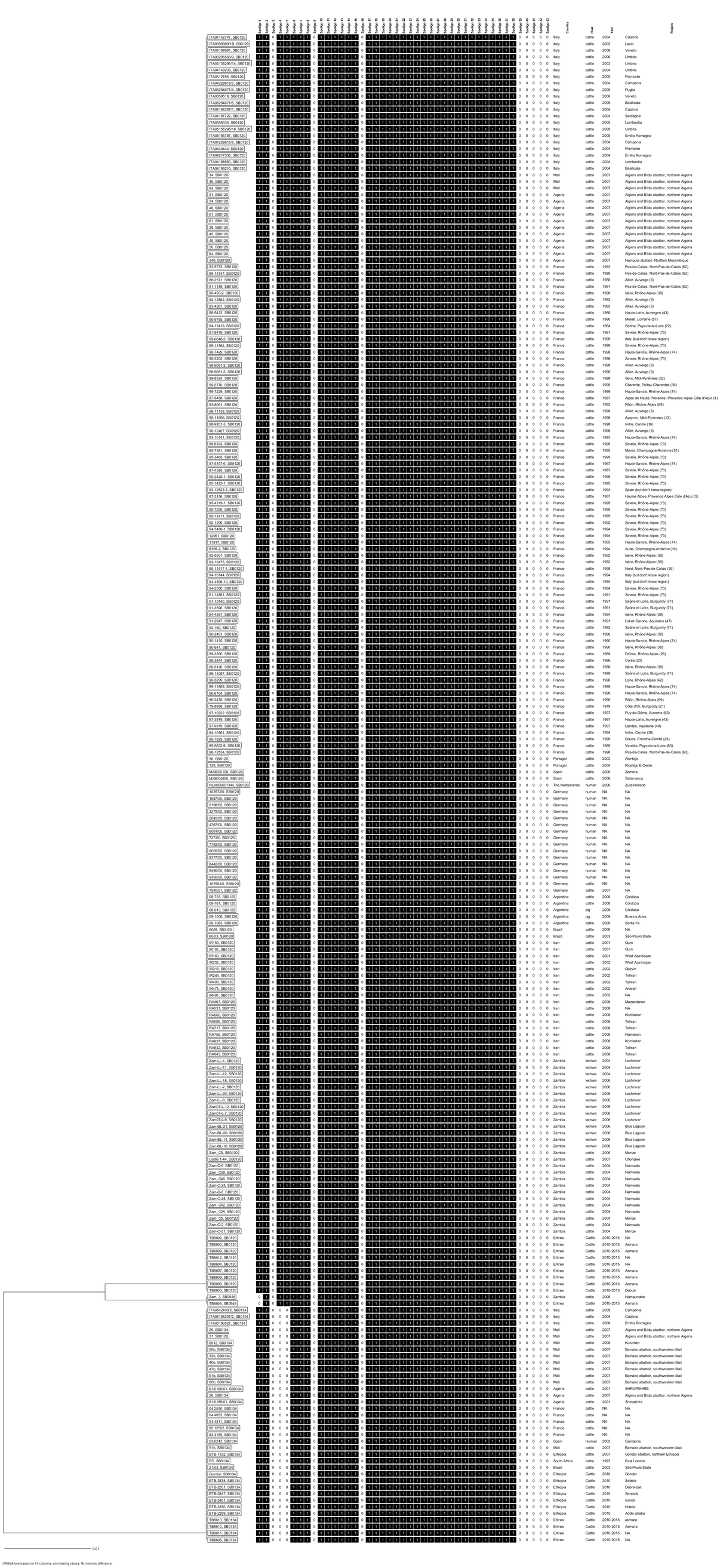

Supplement: S1 Fig — (TIF) [file pntd.0006406.s003.tif]

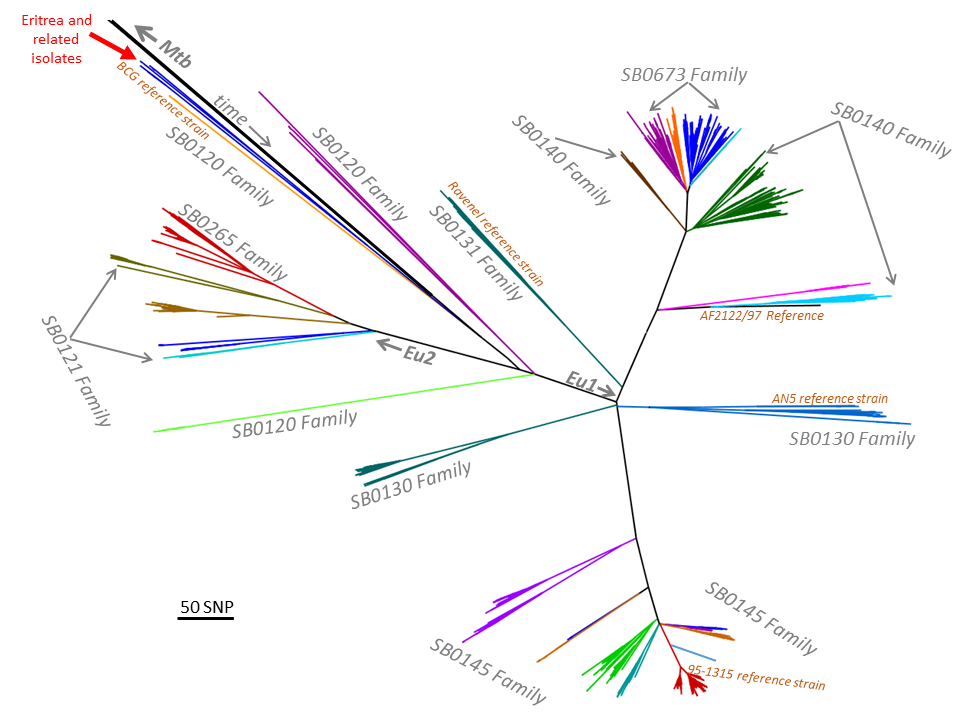

Supplement: S2 Fig — (TIF) [file pntd.0006406.s004.tif]
